# Report of the human body louse (*Pediculus humanus*) from clothes sold in a market in central Italy

**DOI:** 10.1186/s13071-019-3458-z

**Published:** 2019-05-03

**Authors:** Claudio De Liberato, Adele Magliano, Federico Romiti, Michela Menegon, Fabiola Mancini, Alessandra Ciervo, Marco Di Luca, Luciano Toma

**Affiliations:** 1 Istituto Zooprofilattico Sperimentale del Lazio e della Toscana “M. Aleandri”, Via Appia Nuova 1411, 00178 Rome, Italy; 2 Istituto Superiore di Sanità, Viale Regina Elena 299, 00161 Rome, Italy

**Keywords:** *Pediculus humanus*, Body louse, Parasite, Vector, Marketed clothes, Italy

## Abstract

**Background:**

*Pediculus humanus*, the human body louse, is widespread where overcrowding and lack of hygiene are present, in areas of the world affected by poverty, war, famine and presence of refugees. It has recently been considered re-emerging among homeless populations in developed countries. In Italy, it was last reported in 1945. *Pediculus humanus* is a vector of highly relevant human pathogens.

**Methods:**

In October 2018, a woman found small insects on a T-shirt bought second-hand in a local street market in a village 35 km south of Rome (central Italy). Insects were identified both morphologically and by molecular analysis. Moreover, they were analyzed molecularly for the presence of *Rickettsia prowazekii*, *Borrelia recurrentis*, *Bartonella quintana*, *Coxiella burnetii* and *Yersinia pestis*.

**Results:**

Morphological and molecular analyses of the insects identified them as 26 lice (12 females, 10 males and 4 nymphs) of the species *P. humanus*. Many nits were found on the T-shirt seams. DNA of the investigated pathogens was not detected in any of the lice.

**Conclusions:**

The exceptionality of the described case lies both in the report of *P. humanus* from a country where it had not been reported since 1945, and in its finding from second-hand clothes for sale in a market, constituting a potential source of infection for people buying this type of goods. The question arises, how did adults and nits of *P. humanus* infest clothes for sale on a market stall in a country where it had not been reported for decades. Given that the body louse requires frequent blood meals to survive and develop, its arrival on clothes imported from abroad is highly improbable. Hence, it must be presumed that people infected with the human body louse are present in Italy. This report points out a serious regulatory problem regarding the management of second-hand clothes prior to sale and, more generally, of controls in street markets.

## Background

Humans are parasitized by three lice species (Insecta: Anoplura: Pediculidae): *Pediculus humanus*, the body louse; *Pediculus capitis*, the head louse; and *Phthirus pubis*, the pubic louse. Taxonomy within the genus *Pediculus* has been under debate for decades and is still unsolved; depending on the authors, body and head lice can be considered species, subspecies (*Pediculus humanus humanus* and *Pediculus humanus capitis*) or even just ecotypes of the same species [1]. Furthermore, adopting the classification of subspecies, the name of the body louse is controversial, at times being called *P. humanus corporis* or *P. humanus humanus*. In the present work the species will be indicated as *Pediculus humanus*.

*Pediculus capitis* has a worldwide distribution and, along with the nematode *Enterobius vermicularis*, it is the most prevalent human parasite in developed countries, irrespective of the social level, mainly infesting school children and their families [1]. *Pediculus humanus* is widespread where overcrowding and lack of hygiene are present [2]. Common in areas of the world affected by poverty, war, famine and the presence of refugees [3, 4], this species is now considered re-emerging among homeless populations in developed countries [2]; it has recently been reported in France, the Netherlands, Russia and the USA [5–7]. In the literature, the last report of *P. humanus* from Italy is the finding of some specimens on a human in 1945, in Forlì, Emilia Romagna region [8]. In 2015, two cases of louse-borne relapsing fever were reported in Turin, northern Italy, in two refugees living in Italy since 2011 [9], thus highlighting autochthonous transmission. However, the two individuals used to live in an overcrowded refugee hospitality facility, together with newly arrived infected persons; hence, it is presumable that transmission occurred within the facility.

In comparison to *P. capitis* and *P. pubis*, *P. humanus* does not lay its eggs (nits) on the host, but on the seams of their clothes, moving to the host when it seeks a blood meal. Hence, body pediculosis occurs when clothes are not changed or washed regularly in overcrowded and unhygienic environments [2].

*Pediculus humanus* is a vector of important human pathogens, the most relevant being *Rickettsia prowazekii*, the etiological agent of the epidemic typhus, *Borrelia recurrentis*, causing relapsing fever and *Bartonella quintana*, the causative agent of trench fever [3]. These pathogens are not transmitted *via* louse bite, but through the contamination of a bite site or mucous membranes with louse feces or, in the case of *B. recurrentis*, with crushed lice [2, 10]. Currently, louse-borne diseases are mainly reported from central and eastern Africa, the Peruvian Andes, Russia, and in homeless populations from several developed country such as the USA, France and the Netherlands [11–13]. In Italy, as with the vector itself, louse-borne diseases have not been reported for decades, but recently, with the starting of the humanitarian crisis leading thousands of refugees from endemic countries to southern Europe, many imported cases of *B. recurrentis* have been described [9, 10, 14–16].

We hereby describe the first record of *P. humanus* from Italy since 1945, found on second-hand clothes for sale in a street market.

## Methods

### Specimen finding and collection

In October 2018, a woman contacted the Laboratory of Parasitology of the Istituto Zooprofilattico Sperimentale del Lazio e della Toscana “M. Aleandri” of Rome, having found “small, living insects” on a second-hand T-shirt bought two days before in a stall of a local street market in the village of Aprilia, 35 km south of Rome (central Italy). Aprilia Saturday street market is a market where both new and second-hand clothes are sold in stalls, heaped without any kind of packaging. The woman also highlighted that there was a sort of “grey powder” on some parts of the T-shirt, which was difficult to clean out; in her attempt of cleaning the garment, she washed it twice in a washing machine at 40 °C. When the T-shirt was brought to the Laboratory of Parasitology, it was carefully inspected, looking for insects and the “grey powder” with the aid of a stereomicroscope. After morphological identification, recovered insects were analysed molecularly to confirm their specific identification and for the detection of possible pathogens.

### Molecular analyses

#### Lice identification

Total genomic DNA from lice was extracted using PureLink Genomic DNA kits (Invitrogen, Carlsbad, CA, USA) according to the manufacturer’s instructions. DNA from each louse was eluted in 70 µl of elution buffer. The extracted genomic DNA was stored at 20 °C until PCR amplification.

For the molecular identification of louse mitochondrial clades, DNA samples were investigated by PCR-amplification and sequencing of a portion of the mitochondrial gene cytochrome b (*cytb*) using the primers cytbF1 (5′-GAG CGA CTG TAA TTA CTA ATC-3′) and cytbR1 (5′-CAA CAA AAT TAT CCG GGT CC-3′), as described in Raoult et al. [17]. All PCR products were sent to Eurofins Genomics (Ebersberg Germany) for sequencing. The obtained sequences were compiled and analyzed by Accelrys Gene software.

The clades and genotypes of each *cytb* sequence obtained from the analyzed lice were determined by BLASTn search by comparing against sequences available in the GenBank database. For phylogenetic analysis, the neighbor joining method with Tajima-Nei distance [18] was used as a tree-building model using the Accelrys DS Gene software package (Accelrys Inc., San Diego, CA, USA). The newly described sequences from this study were deposited in GenBank under the accession numbers MK248879–MK248904.

#### Pathogen detection

To investigate the presence of bacteria that are highly pathogenic to humans, all genomic DNA samples from lice were individually screened for *B. recurrentis*, *R. prowazekii*, *B. quintana*, *Coxiella burnetii* (Q fever) and *Yersinia pestis* (plague).

A real-time platform was used for the molecular investigation and specific primers and probes used in this study are listed in Table 1. All real-time PCRs were performed in glass capillary tubes (Roche Diagnostics GmbH, Mannheim, Germany) using a Quantifast Probe PCR Kit (Qiagen, Hilden, Germany) and were carried out in a LightCycler 2.0 instrument (Roche Diagnostics GmbH, Mannheim, Germany), with protocols and PCR parameters as previously described [19–23]. The DNA of the target bacteria and master mixes were used as positive and negative controls, respectively.

**Table 1:** Primers and probes used for detection of pathogens in body lice

Organism	Target gene	Primer/probe sequence (5′-3′)	Reference
*Borrelia* spp.	*16S* RNA	AGCCTTTAAAGCTTCGCTTGTAG	[19]
		GCCTCCCGTAGGAGTCTGG	
		FAM-CCGGCCTGAGAGGGTGAACGG-TAMRA	
*Rickettsia prowazekii*	*omp*B	AATGCTCTTGCAGCTGGTTCT	[20]
		TCGAGTGCTAATATTTTTGAAGCA	
		FAM-CGGTGGTGTTAATGCTGCGTTACAACA-TAMRA	
*Bartonella quintana*	*gltA*	GGGGACCAGCTCATGGTGG	[21]
		AATGCAAAAAGAACAGTAAACA	
		GCAAAAGATAAAAATGATTCTTTCCG-Fluorescin	
		LC640-CTTATGGGTTTTGGTCATCGAGT-Phosphate	
*Coxiella burnetii*	*IS1111*	GTCTTAAGGTGGGCTGCGTG	[22]
		CCCCGAATCTCATTGATCAGC	
		FAM-AGCGAACCATTGGTATCGGACGTT-TAMRA-TATGG	
*Yersinia pestis*	*yer*	GCAGGAAATGCGCAATGC	[23]
		GGGCGGATCCCCACTTTA	
		FAM-CG AGG TTC AGG TGA GCA CG-TAMRA	

## Results

### Morphological analyses

**Fig. 1:**
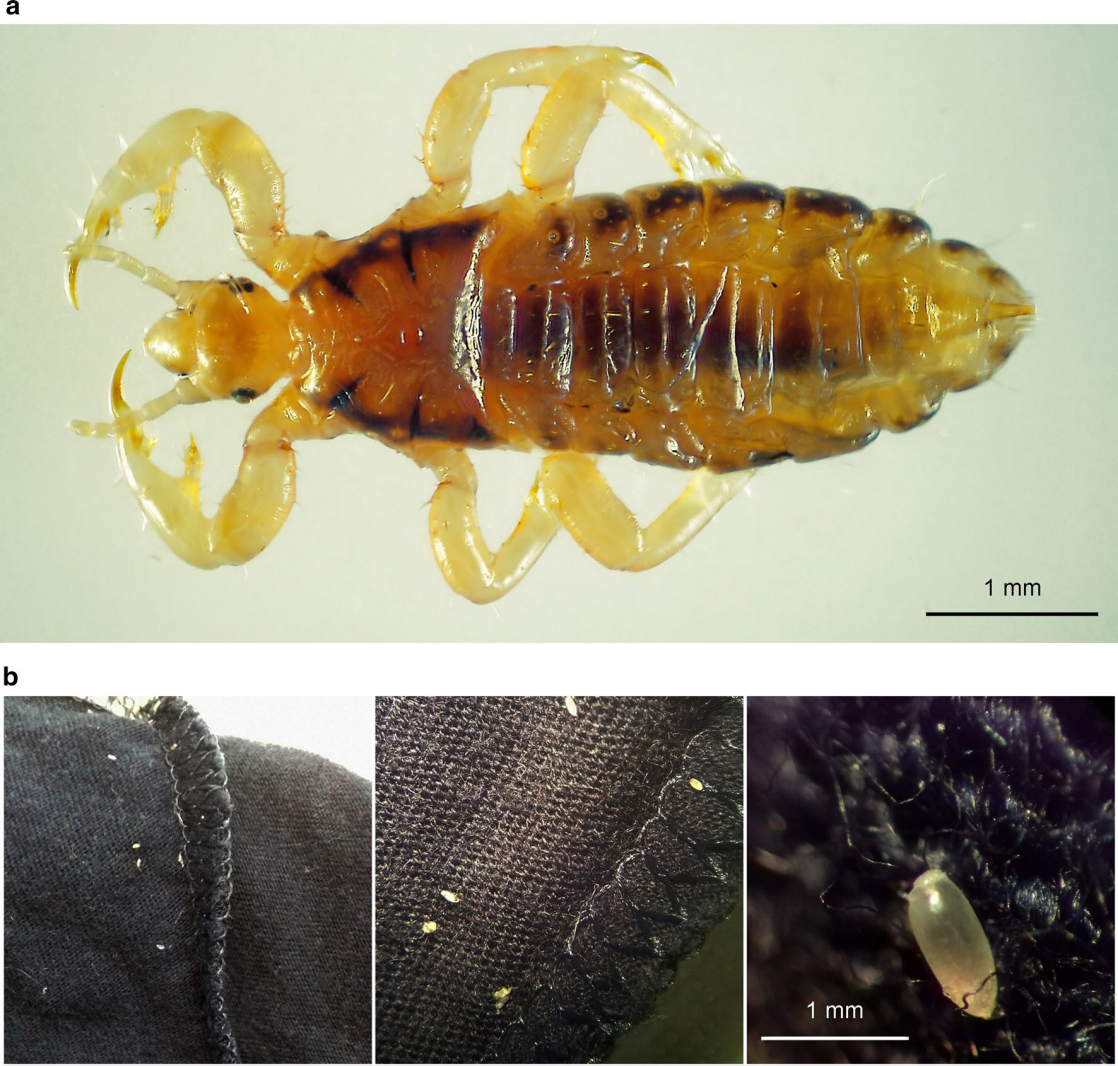
Adult male *Pediculus humanus* (**a**) and several nits attached to the T-shirt seams (**b**)

Upon visual inspection of the T-shirt, it was possible to detect 26 lice (12 females, 10 males and 4 nymphs), morphologically ascribable to the genus *Pediculus* (Fig. 1a). Even though the T-shirt had been washed twice in a washing machine, lice were still alive at the moment of inspection. What was described by the woman as a grey powder, under the stereomicroscope was determined to be louse nits (Fig. 1b), numerous along the seams of the T-shirt, especially near the armpit. The report of lice on an item of clothing and the finding of nits attached to seams led us to identification of the lice as *P. humanus*, the human body louse. Lice morphology and size (up to 7 mm in length for females) were compatible with this identification [24, 25].

### Molecular analyses

**Fig. 2:**
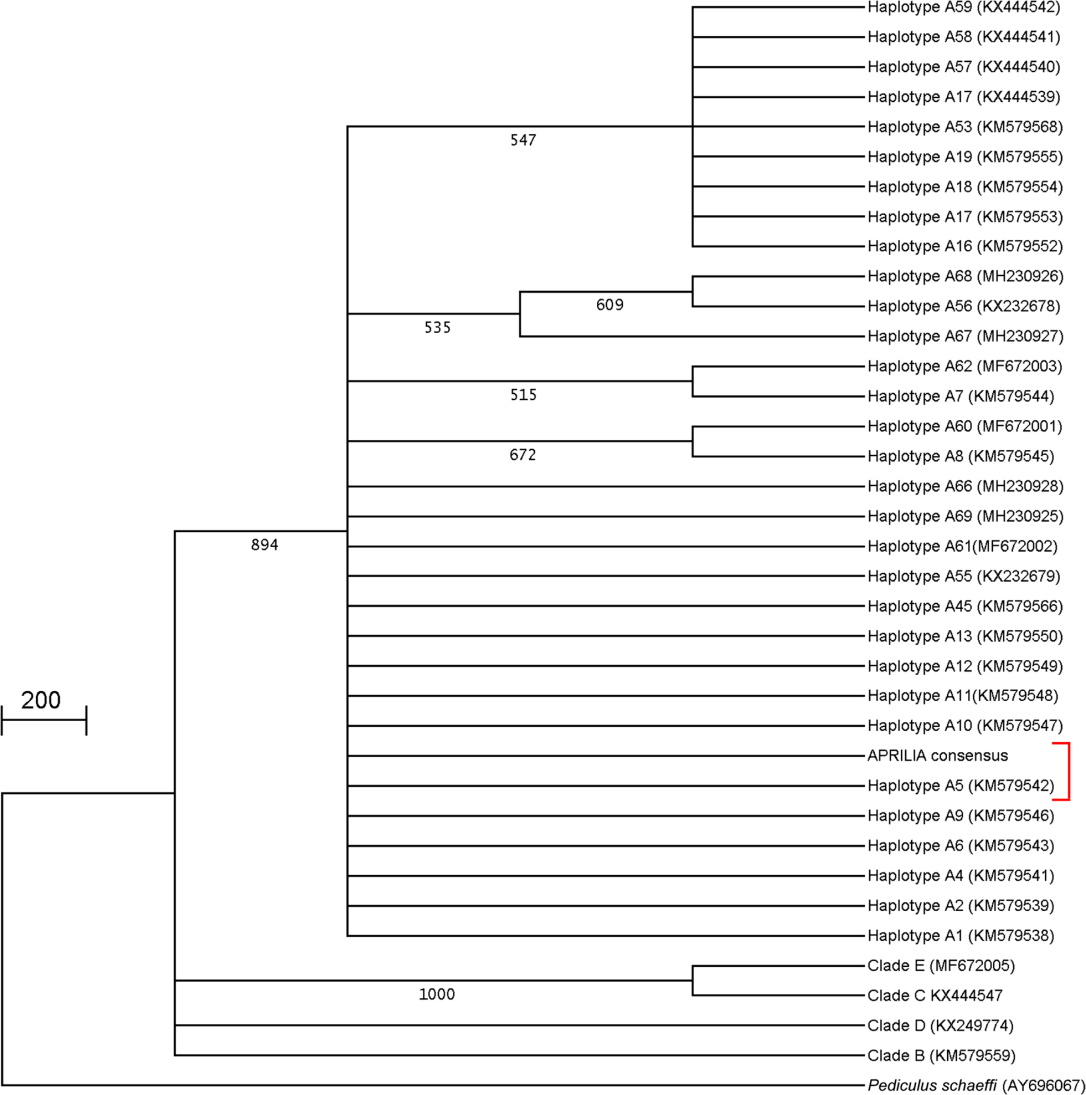
Phylogenetic analysis of cytochrome c oxidase sequences (272 bp) belonging to consensus sequence obtained from Aprilia lice and all representative haplotypes within the clade A, identified so far, retrieved from the GenBank database. Phylogenetic trees of alignment were constructed using the neighbor-joining method by bootstrapping with 1000 replicates, and phylogenetic distances were measured by Tajima–Nei model. Only values > 50% are given. Numbers at nodes indicate the level of bootstrap support. Four sequences representative of the haplotypes B, C, D and E were also included in the analysis. A chimpanzee louse *Pediculus schaeffi* was used as an outgroup

A 348-bp region of the mitochondrial cytochrome *b* gene (*cytb*) from all 26 lice was sequenced. No intraspecific variability was detected, and the resulting Aprilia consensus sequence showed 100% identity with a previously described haplotype A5 (KM 579542) belonging to the louse clade A [26]. To analyze phylogenetic relationships, a phylogenetic tree was built based on alignment of a portion of 272 nucleotides of cytb region between the Aprilia lice consensus sequence and 31 *P. humanus* sequences from clade A available in GenBank. In addition, four sequences representative of haplotypes B, C, D and E and a sequence of *Pediculus schaeffi*, as outgroup (accession no. AY695999), were included in the analysis (Fig. 2).

The phylogenetic tree confirmed that the Aprilia consensus sequence clearly belongs to clade A, sharing a total nucleotide identity with haplotype A5, the most common haplotype worldwide, and differing from one to four nucleotides with the 14 closest haplotypes.

DNA of the five investigated pathogens was not detected in any of the examined lice.

## Discussion

The exceptionality of the described case lies not only in the report of *P. humanus* from a developed country (Italy) where it had not been reported for decades, but also in its report from second-hand clothes for sale in a market, constituting a potential source of infection for people buying such goods and thus possibly spreading this parasite out of the typical host range where it is presently found in developed countries, homeless people and refugees [3, 4].

The following question therefore arises: how did adults and nits of *P. humanus* infest a garment for sale on a market stall in a country where it had not been reported for many years? Given that *P. humanus* need frequent blood meals each day to develop and survive, it has to be presumed that lice found alive on the T-shirt were able to feed in the few hours preceding their discovery [3], thus excluding their arrival from abroad on imported clothing. Unfortunately, since it was impossible to pinpoint the exact stall where the T-shirt was bought, a thorough investigation could not be made.

For the peculiar mode of louse-borne disease transmission, occurring through contamination of clothes with lice feces or crushed specimens [3], in the present case the risk of transmission does not seem to be negligible. As a matter of fact, people searching for clothes in the heaps on the stalls could theoretically be exposed to infection, contaminating their hands with infected material, without buying or wearing the infected item. Lice on the T-shirt were alive at the moment of inspection, irrespective of the two washings at 40 °C. This confirms findings on the sanitation of clothes harbouring body lice and its nits, whereby a temperature of at least 60 °C is required to kill lice and nits [10].

The attribution of lice to haplotype A5 was expected, being the more commonly detected clade with a worldwide distribution [1, 26, 27]. Consequently, this finding does not allow for speculation on their possible geographical origin. Despite the fact that none of the five main louse-borne pathogens were detected in the analyzed lice, recent human cases of *B. recurrentis* occurring in Italy have raised concerns in the health authorities about the real risk of this and other infective agents being introduced through migrant flows [10, 14–16]. For this purpose, further screening of other louse-borne pathogens has been planned.

## Conclusions

*Pediculus humanus*, an important human parasite and vector, previously considered extinct in Italy, is unexpectedly found present in the country. Given that the last report was over 70 years ago and the generally high level of hygiene in Italy, body lice have so far been considered something of the past; this report is presumably the result of a re-introduction. For the type of report, an infested garment for sale in a market, we can probably exclude that the source of infestation was homeless people, typically considered the most at-risk category for this kind of parasite in developed countries [2]. Living lice arrived alive on second-hand clothes for sale in a street market due to the inappropriate management of goods (i.e. not subjected to thorough sanitation before sale) and to the very short time interval between use by the previous owner and its sale. This report points out a serious regulatory problem regarding the management of second-hand clothes prior to sale and, more generally, of controls in street markets.

## Abbreviations

PCR: polymerase chain reaction
*Cytb*: mitochondrial gene cytochrome *b*
